# Smile – the next generation of laser vision correction

**Published:** 2016

**Authors:** Nicolae Miruna, Filip Andrei, Filip Mircea Vasile, Rotaru Eugen

**Affiliations:** * AmaOptimex, Eye Clinic, Bucharest, Romania

**Keywords:** SMILE, lenticule, myopia, Femtolaser

## Abstract

Our paper is an introduction in this new generation of Laser vision correction, called SMILE. It also reveals our experience in the past year, since we started to perform this new procedure in our patients. Small Incision Lenticule Extraction technique is the 3rd generation of Laser vision correction that completely redefines refractive surgery. Being performed entirely with femtosecond laser, SMILE is tissue preserving and very gentle for the eye. In 2011, it was launched internationally.

We have started with SMILE in October 2014. Since then, we have performed more than 200 procedures, with the range of corrected diopters between -2 and -10 and astigmatism between -2 and -5. In the near future, hyperopic diopters will be corrected with SMILE.

## Introduction

Professor Walter Sekundo first performed ReLex in 2006. ReLEX, refractive lenticule extraction, includes SMILE (Small incision lenticule extraction) and FLex (femtosecond lenticule extraction - initial form of ReLEX). More than 250 000 SMILE procedures have been performed all over the world since 2011, when it was launched internationally.

SMILE is entirely performed with femtosecond laser, it is a minimally invasive, flapless surgery, and redefines refractive surgery as we know it. SMILE is the 3rd generation of corneal refractive procedures, as Dr. Rupal Shah was describing it: “SMILE is a LASIK without a flap and a PRK without pain”. It is the only laser refractive surgical technique that can correct high diopters of myopia+/ -myopic astigmatism, up to 10 D (spherical equivalent).

## Laser System and the inflammatory response

ReLEX SMILE is exclusively performed with one laser, a femtosecond laser that ensures high- level reproducibility and predictability, even with high corrections [**1**].

The VisuMax system uses lower pulse energy and higher pulse frequency (500 kHz). A lower pulse energy is generally associated with fewer unwanted side effects (opaque bubble layer = OBL, collateral thermal damage, corneal inflammation, diffuse lamellar keratitis) [**1**].

The inflammatory response and wound healing after SMILE is minimal and subsides after one week postoperatively. SMILE induces less keratocyte apoptosis, proliferation, and inflammation compared to FemtoLASIK [**1**,**2**]. Corneal haze and epithelial ingrowth have been reported to be low, and mainly associated with difficult lenticule extraction. The corneal wound-healing and inflammatory response has an important impact on the postoperative outcomes. Studies conducted on mice regarding the correlation between the early inflammatory response and the degree of myopia corrected with SMILE, showed that a greater keratocyte response was seen in high myopic corrections [**3**]. Most of the ReLEX surgeons reported an early inflammatory response during the learning curve of SMILE, which was more important in the first cases.

## The procedure

Zeiss Visumax femtosecond laser system cuts the lenticule (first posterior plane then side cuts and afterwards anterior plane), followed by a small incision (3,93 mm) that can be adjusted according to the surgeon’s wishes and experience. The lenticule is removed through a small incision, by passing a dissector, and, the anterior and posterior lenticular interfaces are separated. This eliminates the need to create a flap, and the cornea above the upper interface of the lenticule, is called cap [**1**]. The corneal caps of SMILE are predictable with good reproducibility, regularity, and uniformity. Cap morphology might have a mild effect on the refractive outcomes in the early stage [**3**].

The ReLEX SMILE procedure is performed under topical anesthesia and it can be divided into two steps: the femtosecond laser application and the manual removal of the lenticule [**1**]. It can be performed bilaterally, either as two sequential procedures or the first laser applied on both eyes and then the lenticules are extracted [**4**]. By removing the lenticule, the cornea’s shape is changed, thereby achieving the desired refractive correction.

The laser procedure lasts for about 25 seconds and the extraction of the lenticule for another couple of minutes. The corneal cap thickness is of 120 microns, the residual stroma of 250 microns and the lenticule diameter is of 6,5 mm.

**Fig. 1:**
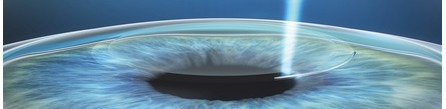
Lenticule creation in SMILE technique

## Indications and advantages

SMILE is used to correct myopia of up to -10 diopters and myopic astigmatism of up to -5 diopters or combined of up to -10 diopters.

SMILE’s advantages for patients: Safe: no flap, therefore no flap complications and preservation of the corneal nerves gets a less dry eye syndrome; greater integrity of the upper corneal layers, preserving the biomechanical stability of the cornea; gentle for the eye; tissue preserving; painless; quick recovery, low regression rate [**1**,**4**]. It is an odorless and noiseless laser procedure.

SMILE’s advantages for doctors: fast procedure and no patient relocation, therefore it is possible to treat more patients in less time. It offers an optimized workflow. Only one laser is used, one treatment planning and one laser procedure. Excellent clinical outcomes have been proven by the studies and the surgeons’ experiences. SMILE provides a differentiation and a new premium procedure. SMILE provides the WOW factor.

## Our experience

We have started with SMILE in October 2014, and, since then, we have performed more than 200 procedures. The range of the corrected diopters was between -2 and -10 and of astigmatism between -2 and -5. The learning curve might be challenging because of the novelty, but, being an experienced surgeon, also helps a lot.

While preparing for the surgery, the patients follow a complete ocular examination including: biomicroscopy, refraction and keratometry, cycloplegic refraction, intraocular pressure, Schirmer test, corneal topography, Pentacam (to scan any form frusta keratoconus), pachymetry, Humphrey perimetry, ocular ultrasound and biometry with both IOL Master and Ultrascan. The keratometry is performed with the refractometer, topographer, and IOL Master. Afterwards, a long series of explanations, regarding the procedure and a preoperative treatment with topical antibiotics follows two days before surgery [**5**].

After the procedure, topical antibiotics are prescribed for a week, topical steroids for three weeks, with a higher dose for high myopias, being tapered gradually, and artificial tears for three weeks. The patients’ examination is performed: the next day, after one week, a month, 3 months, 6 months and one year.

The procedure is very well tolerated by the patients. The most disturbing secondary reaction postoperatively is the blurred vision, which lasts for a few hours. Very few patients experienced discreet tearing and photophobia for a couple of hours after the surgery.

Very good results have been achieved so far. A slow rate of visual acuity recovery was obtained in 7 eyes due to the stromal reaction. Five patients had problems readapting to reading and writing activities due to the perception changes, and, the de-epithelization of the cornea close to the incision appeared in 6 cases, having to use a contact lens to increase the comfort of the patient.

## Conclusions

**Fig. 2:**
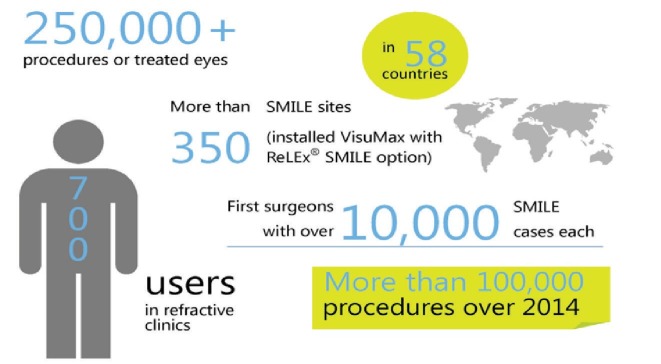
SMILE and numbers

**Fig. 3:**
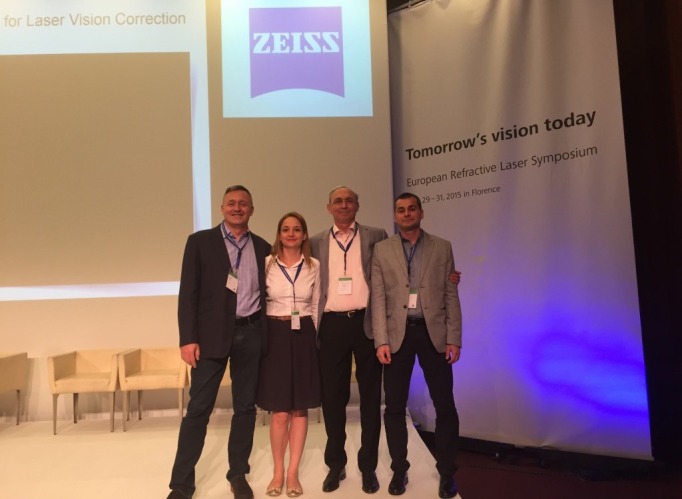
Romanian SMILE team

SMILE is the 3rd generation of laser refrac- tive surgery technique available for myopic pa- tients, but attempts are also made to treat higher myopia and also hyperopia. The development of SMILE suggests that the future of refractive sur- gery is minimally invasive.

AMA Optimex Clinic receives educational support from Carl Zeiss Instruments SRL; there are no other financial interests involved.
